# N‐Glycan profiling of chondrocytes and fibroblast‐like synoviocytes: Towards functional glycomics in osteoarthritis

**DOI:** 10.1002/prca.202000057

**Published:** 2021-03-12

**Authors:** Johannes Fuehrer, Katharina M. Pichler, Anita Fischer, Alexander Giurea, Daniela Weinmann, Friedrich Altmann, Reinhard Windhager, Hans‐Joachim Gabius, Stefan Toegel

**Affiliations:** ^1^ Department of Chemistry University of Natural Resources and Life Sciences Vienna Austria; ^2^ Karl Chiari Lab for Orthopaedic Biology Department of Orthopedics and Trauma Surgery Medical University of Vienna Vienna Austria; ^3^ Ludwig Boltzmann Institute for Arthritis and Rehabilitation Vienna Austria; ^4^ Department of Orthopedics and Trauma Surgery Division of Orthopedics Medical University of Vienna Vienna Austria; ^5^ Faculty of Veterinary Medicine Institute of Physiological Chemistry Ludwig‐Maximilians University Munich Munich Germany

**Keywords:** chondrocytes, galectins, glycomics, osteoarthritis, sialylation

## Abstract

**Purpose:**

N‐Glycan profiling provides an indicator of the cellular potential for functional pairing with tissue lectins. Following the discovery of galectin expression by chondrocytes as a factor in osteoarthritis pathobiology, mapping of N‐glycans upon their phenotypic dedifferentiation in culture and in fibroblast‐like synoviocytes is a step to better understand glycobiological contributions to disease progression.

**Experimental design:**

The profiles of cellular N‐glycans of human osteoarthritic chondrocytes and fibroblast‐like synoviocytes were characterized by mass spectrometry. RT‐qPCR experiments determined mRNA levels of 16 glycosyltransferases. Responsiveness of cells to galectins was quantified by measuring the mRNA level for interleukin‐1β.

**Results:**

The shift of chondrocytes to a fibroblastic phenotype (dedifferentiation) is associated with changes in N‐glycosylation. The N‐glycan profile of chondrocytes at passage 4 reflects characteristics of synoviocytes. Galectins‐1 and ‐3 enhance expression of interleukin‐1β mRNA in both cell types, most pronounced in primary culture. Presence of interleukin‐1β leads to changes in sialylation in synoviocytes that favor galectin binding.

**Conclusions and clinical relevance:**

N‐Glycosylation reflects phenotypic changes of osteoarthritic cells in vitro. Like chondrocytes, fibroblast‐like synoviocytes express N‐glycans that are suited to bind galectins, and these proteins serve as inducers of pro‐inflammatory markers in these cells. Synoviocytes can thus contribute to disease progression in osteoarthritis in situ.

**Abbreviations**
FLS: fibroblast‐like synoviocytes
Gal: galectin
IL‐1β: interleukin‐1β
mRNA: messenger RNA
OA: osteoarthritis
p0/p4: passage 0/passage 4
RT‐qPCR: quantitative reverse transcription polymerase chain reaction
TNF‐α: tumor necrosis factor‐α

## 1 INTRODUCTION

Glycophenotyping, the profiling of glycans on cells and in tissues, has initially had a purely descriptive character. However, the growing realization that carbohydrates serve as third alphabet of life and form molecular messages, which are turned into respective bioactivity by pairing with tissue lectins, has added a new functional dimension to their presence [1, 2]. When reprogramming of glycan synthesis is encountered in pathophysiological processes, this could indeed be part of an intimately orchestrated co‐regulation with cognate lectins (e.g., in inflammation together with the three selectins) [3, 4]. Intriguingly, distinct aspects of protein glycosylation can even become switches for regulating cellular gene expression. This has recently been shown in vitro for an association between sialylation and transcriptional activity of genes maintaining breast cancer pathogenicity such as the epidermal growth factor receptor, CD44 or nucleolin [5]. These lines of evidence for clinically relevant glycan functionality give incentive to approach the study of glycan profiles from a new perspective, especially in the context of a common disease such as osteoarthritis (OA).

OA is considered a degenerative disease of the entire joint, involving all joint constituents (cartilage, meniscus, subchondral bone, synovial membrane and infrapatellar fat pad), with unknown etiology. Based on the hypothesis of a functional glycan‐receptor (lectin) interplay in OA, glycophenotyping of human chondrocytes in primary culture has guided us to identify a new class of pathogenic effectors in OA, that is, galectins [6, 7]. Upregulation of expression and the extracellular availability of galectins‐1, ‐3 and ‐8 (Gal‐1, ‐3, ‐8) was then shown to induce a pro‐degenerative and ‐inflammatory gene signature in chondrocytes, with the galectins acting together as a team [8, 9, 10]. Considering the potential of chondrocytes from culture for cartilage regeneration [11] together with the related problem of cellular transition to a fibroblast‐like phenotype after passaging in vitro [12, 13], it is now timely to define any alterations in N‐glycans within this process. Equally important, it is also warranted to include another cell type involved in joint degeneration processes, that is, fibroblast‐like synoviocytes (FLS). Of note, synovial fluid cells secrete a galectin (Gal‐8) that associates with the glycoprotein CD44vRA and hereby affects the local inflammatory reaction in rheumatoid arthritis [14]. Starting with glycan mapping of FLS, we intended to learn more about the potential of cells of the synovial tissue to drive OA progression via glycan‐dependent processes.

Thus, this study aimed to investigate whether (i) the phenotypic change of OA chondrocytes to dedifferentiated fibroblast‐like cells during passaging to p4 is associated with alterations of N‐glycosylation and thus responsiveness to galectins, (ii) there is a similarity in N‐glycosylation between OA chondrocytes (at p0 and at p4) and OA FLS, (iii) a similarity exists between OA FLS and immortalized human synovial fibroblasts (cell line K4IM), (iv) N‐glycosylation in OA FLS is affected by pro‐inflammatory mediators, and (v) OA FLS are responsive to galectins (to a similar extent as chondrocytes at p4 and in primary cell isolates).

## 2 MATERIALS AND METHODS

### 2.1 Cell culture

Clinical specimens of articular cartilage and synovial tissue were obtained from OA patients (13 female, 13 male; age range: 47–87 years; Knee Society Scores: Knee Score 16–63, Functional Score 0–70) during total knee replacement (TKR) surgery with written informed consent and in accordance with the terms of the ethics committee of the Medical University of Vienna (1822/2017 and 1555/2019). Inclusion criteria were end‐stage OA scheduled for TKR and age above 20 years. Exclusion criteria were presence or history of inflammatory or septic arthritis, psychological inabilities or difficulties to be instructed. Comorbidities in included patients comprised hypertension (62%), obesity (38%), cardiovascular diseases (35%), pulmonary diseases (31%), nicotine abuse (27%), hyperlipidemia (19%), hyperuricemia (12%), and diabetes (8%). OA FLS (n = 14 patients) were isolated according to published protocols [15]. For most experiments, OA FLS were used at p4, when confluent monolayers appeared to be fibroblast‐like and were negative for CD68 [16]. A separate experiment analyzed the primary cell isolate from synovial tissue systematically between p0 and p4 (n = 3 patients). K4IM cells (immortalized human synovial fibroblasts, kindly provided by Dr. Favero and Dr. Belluzzi) [17, 18] were maintained in DMEM supplemented with 10% fetal bovine serum and used at 100% confluency at p36–p38. Human OA chondrocytes were isolated from femoral condyles and tibial plateaus of eight patients, from whom OA FLS were also isolated (see above), and cultured following established protocols to allow direct comparison between the two cell types [19]. Chondrocytes of five additional patients were independently taken to p4 to allow direct comparison of OA chondrocytes in p0 and p4.

**Clinical Relevance**

The emerging role of tissue lectins (galectins) in the progression of osteoarthritis directs interest to the profiling of N‐glycans in osteoarthritic cells. In addition to chondrocytes, fibroblast‐like synoviocytes are capable to contribute to galectin‐elicited dysregulation of functional disease markers. Our study reveals that distinct aspects of their N‐glycosylation (i.e., α2,3/6 sialylation) are shifted by interleukin‐1β towards enhanced ligand activity with galectins. Moreover, the data point to a potential functional relevance of the shift among N‐glycans and can thus help to identify new target sites for innovative therapeutic interventions in osteoarthritis on the level of glycan ligands for disease‐associated galectins.

### 2.2 RT‐qPCR measurements

Total RNA isolation, cDNA synthesis and SYBR‐green‐based RT‐qPCR experiments (including details on primer sequences and efficiencies) had previously been described [6]. The protocols followed the minimal guidelines for the design and documentation of qPCR experiments [20]. mRNA expression levels for 16 glycosyltransferases involved in N‐glycan processing and maturation at different stages from the conversion to hybrid‐ and complex‐type structures to α2,3/6‐sialylation were calculated as relative copy numbers with respect to the geometric mean of the expression of glyceraldehyde‐3‐phosphate dehydrogenase (GAPDH), β‐actin (ACTB) and succinate dehydrogenase complex, subunit A (SDHA) arbitrarily set to 1000. The effect of interleukin‐1β (IL‐1β) or galectins on mRNA levels was quantified as fold changes relative to untreated cultures, considering normalization to GAPDH.

### 2.3 Stimulation of cells with cytokines and galectins

At 90% confluency, cell cultures were serum‐starved overnight and treated for 24 h (RT‐qPCR) or 5 days (mass spectrometry) with human recombinant IL‐1β (10 ng/mL) or tumor necrosis factor‐α (TNF‐α) (40 ng/mL) (both from Biolegend) to induce an aspect of pro‐inflammatory conditions. In another set of experiments, serum‐starved cells were treated with 10 or 50 μg/mL human Gal‐1 or Gal‐3 for 24 h, prior to RT‐qPCR analysis. Recombinant Gal‐1 and ‐3 were prepared, purified, checked for maintained activity and tested under conditions as described previously [8, 9]. Control cultures of cells from the same patient were processed in parallel.

### 2.4 Sample preparation for mass spectrometry (MS)

Adherent cells were washed thoroughly with phosphate‐buffered saline to remove any components of the culture medium. Cells were lysed with 100 mM ammonium bicarbonate solution (Sigma‐Aldrich) containing 2% SDS (Bio‐Rad). Dithiothreitol (Sigma‐Aldrich) was added to the solution to a concentration of 30 mM, the sample was incubated for 5 min at 95°C followed by another 30 min at 56°C. Addition of iodoacetamide (Sigma‐Aldrich) to a final concentration of 75 mM followed, and the mixture was incubated for 30 min in the dark at room temperature. Then, the samples were centrifugated at 10,000 rcf for 5 min to remove cell debris, the resulting supernatant was treated with chloroform/MeOH for protein precipitation [21].

This material was dissolved in 99 μL of 50 mM ammonium acetate buffer at pH 8.4. 1 μL of solution containing N‐glycosidase F (1 U/μL; Roche) was added, the mixture incubated for 16 h at 37°C. Prior to starting N‐glycan purification, the reaction was stopped with 800 mM ammonium formate buffer at pH 3. The N‐glycans were separated from proteins with C18 solid‐phase extraction cartridges (Thermo Fisher) according to the manufacturer's protocol. The free N‐glycans from the flow‐through fraction were reduced with 1% NaBH_4_ (Sigma‐Aldrich) in 50 mM NaOH for 16 h at room temperature, products were then processed with PGC solid‐phase extraction cartridges (Thermo Fisher) according to the manufacturer's protocol. Glycans were eluted with 55% acetonitrile in 100 mM ammonium formate buffer at pH 3, dried and then dissolved in HQ‐H_2_O.

### 2.5 N‐Glycan profiling by MS

All samples were measured in positive mode with a quadrupole time‐of‐flight (Q‐TOF) instrument (maXis 4G; Bruker). Standard source settings (capillary voltage 4.5 kV, nebulizer gas pressure 0.5 bar, drying gas 5 L/min, 200°C) were used. For a run, purified samples were loaded on a PGC column (100 mm x 0.32 mm, 5 μm; Thermo Fisher Scientific) using 80 mM ammonium formate buffer of pH 3.0 as aqueous solvent. A linear gradient from 1% solvent B (80% acetonitrile plus 20% solvent A) to 65% solvent B in 39 min was applied, at a flow rate of 6 μL/min. Detection was performed with the Q‐TOF instrument equipped with a standard ESI source in data‐dependent acquisition mode (switching to MS/MS mode for eluted peaks) that is directly linked to the Thermo Ultimate 3000 UPLC system. MS scans were recorded within a range of 150 to 2200 Da. Instrument calibration was performed with an ESI calibration mixture (Agilent).

Initial data processing was done with DataAnalysis 4.0 and QuantAnalysis 2.0 (Bruker). Known differences in the retention time during PGC‐separation identified the type of sialylgalactose linkage [22]. Due to inherent ambiguity with N‐glycans harboring three or more antennae, linkage positions were only determined for biantennary structures.

### 2.6 Statistical evaluation

Statistical analyses of RT‐qPCR data were performed using IBM SPSS 25.0. Normal distribution of the data was analyzed using the Shapiro‐Wilk test. Statistical significance of normally distributed data was delineated using paired Student's *t*‐test, whereas non‐normally distributed data were analyzed using the Wilcoxon signed rank test. *p*‐Values < 0.05 were considered significant.

MS data were processed using the version 3.7 of the python programming language (Python Software Foundation, https://www.python.org/), the “pandas” package (version 1.0.3) for general data handling and the “SciPy” package (version 1.4.1) for statistical calculations. The relative abundance of each glycan was calculated and tested for normal distribution using the Shapiro‐Wilk test using a significance level of 0.1. Depending on the nature of the distribution, either a paired Student's *t*‐test or a Mann‐Whitney rank test was used to answer the question whether two distributions differ from each other. In this case, a significance level of 0.05 was used. The Benjamini‐Hochberg procedure was additionally applied to correct for type I errors caused by multiple hypothesis testing at a false discovery rate of 5%.

Relative abundance data were first transformed to standard scale and reduced to those two dimensions, which contribute the most to the variance in a sample. MS data was plotted using the “seaborn” package version 0.10.1. Heatmaps were generated using relative abundance data to calculate the Euclidean distance matrix, applying a hierarchical clustering algorithm. The data were standardized among all plotted samples for each individual type of N‐glycan.

## 3 RESULTS

### 3.1 N‐Glycosylation of OA chondrocytes at p0/p4

**FIGURE 1:**
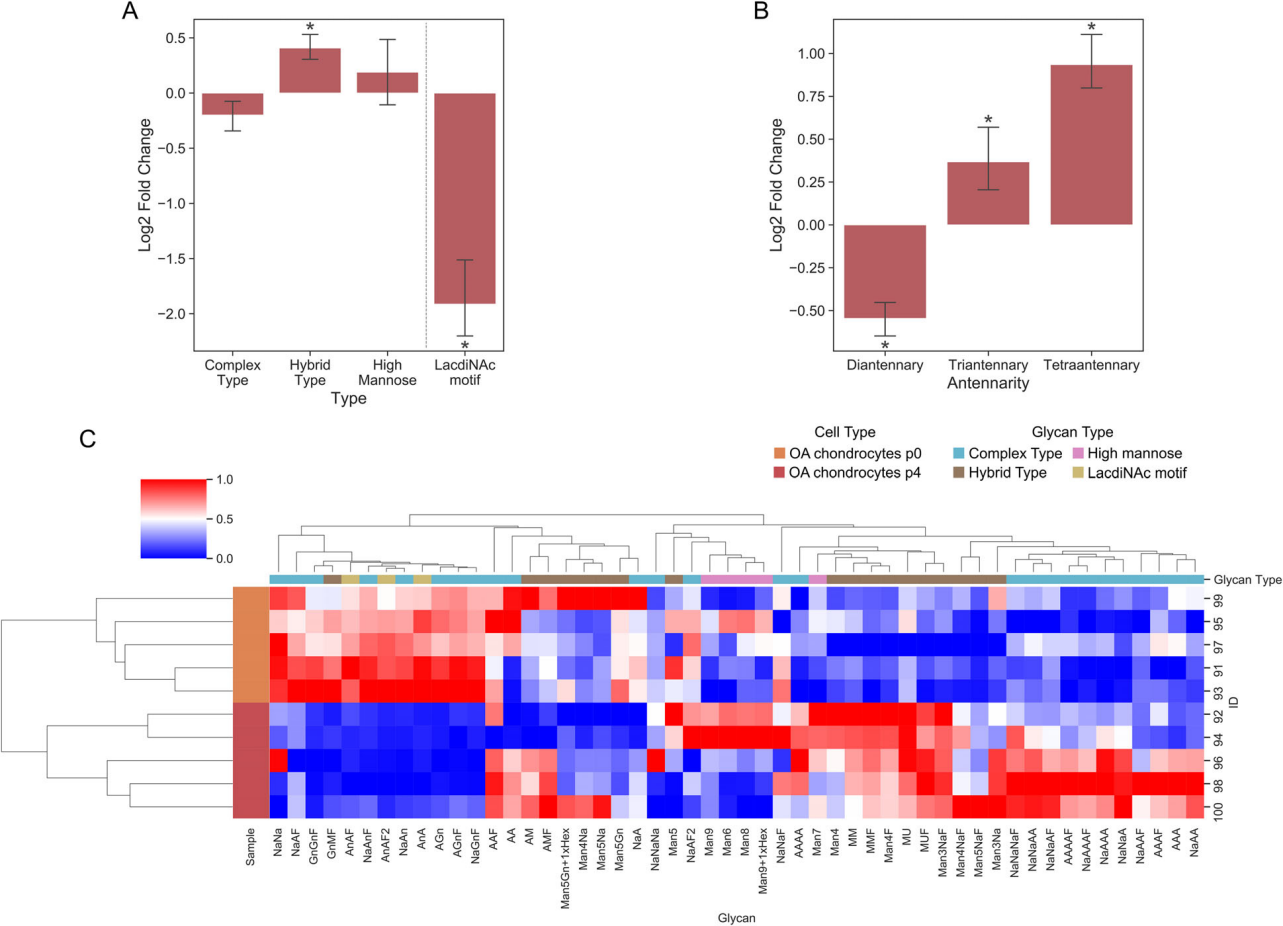
Comparison between the glycophenotypes of OA chondrocytes in p0 and p4. Cell cultures of OA chondrocytes p0 were established from cartilage tissues of five patients, and OA chondrocytes p4 were generated by subsequent passaging of the same cultures. (A,B) Log2 fold change of specific characteristics of N‐glycans, including (A) the glycan type and (B) antennarity of complex‐type structures, in OA chondrocytes after repeated passaging (n = 5 patients). Significant differences to OA chondrocytes p0 are indicated with asterisks (*p < 0.05; n = 5; paired two‐sided *t*‐test or Wilcoxon test). (C) Heatmap showing the hierarchical clustering of the relative glycan abundance in OA chondrocytes at p0 (n = 5) and at p4 (n = 5)

In a previous study, we presented a survey on 21 N‐ and 3 mucin‐type O‐glycans of OA chondrocytes in primary culture, providing first data on the distribution of glycans among the different structural categories [6]. Of note, we found evidence for the presence of possible galectin‐binding structures, defined as “core‐substituted, α2,3/6‐sialylated N‐glycans, with the presence of GalNAcβ1,4GlcNAc (LacdiNAc)‐terminated structures and core 2 O‐glycans” [6]. In order to determine the influence of cell passaging (that leads to the acquisition of a fibroblast‐like morphology) on N‐glycosylation, we comparatively studied chondrocyte populations at p0 and at p4. The data on N‐glycan profiling by MS are presented in the categories of the three classes of the N‐glycans, including the presence of the terminal LacdiNAc structure (Figure 1A), and showing the number of antennae for the complex‐type N‐glycans (Figure 1B). Other classes of N‐glycans (e.g., bisecting or fucosylated structures) are not shown, because no significant differences were detected in pairwise analyses. Additionally, a heatmap‐style illustration summarizing information on the different isobaric structures is presented in Figure 1C. Significant shifts towards hybrid‐type N‐glycans and an increase in the number of glycan antennae in complex‐type N‐glycans were revealed. Slight decreases concerned the status of sialylation, whereas a marked reduction occurred for the presence of LacdiNAc (Figure 1A,C). The highly significant downregulation of the transferase responsible for completing the synthesis of this glycan epitope (B4GALNT3; *p* = 0.003, Table S1) points to an involvement of transcriptional activity of a glycogene in this case of modified glycan production.

The documented changes may have a bearing on cell reactivity for galectins. When testing the responsiveness of both types of OA chondrocyte populations to Gal‐1 or Gal‐3, using production of IL‐1β‐specific mRNA as indicator, the p4 cells turned out to exhibit a significantly decreased response relative to p0 chondrocytes (Figure S1). Passaging of OA chondrocytes thus has an impact on a distinct aspect of their N‐glycan profile and hereby on the capacity of two galectins to trigger their typical effect on the expression of a functional disease marker. Monitoring of N‐glycans was next performed for OA FLS (at p4) to facilitate the comparison to the profile of chondrocytes, hereby examining whether the phenotypic similarity of p4 OA chondrocytes to OA FLS is reflected by this aspect of cell biochemistry.

### 3.2 N‐Glycosylation of OA FLS

**FIGURE 2:**
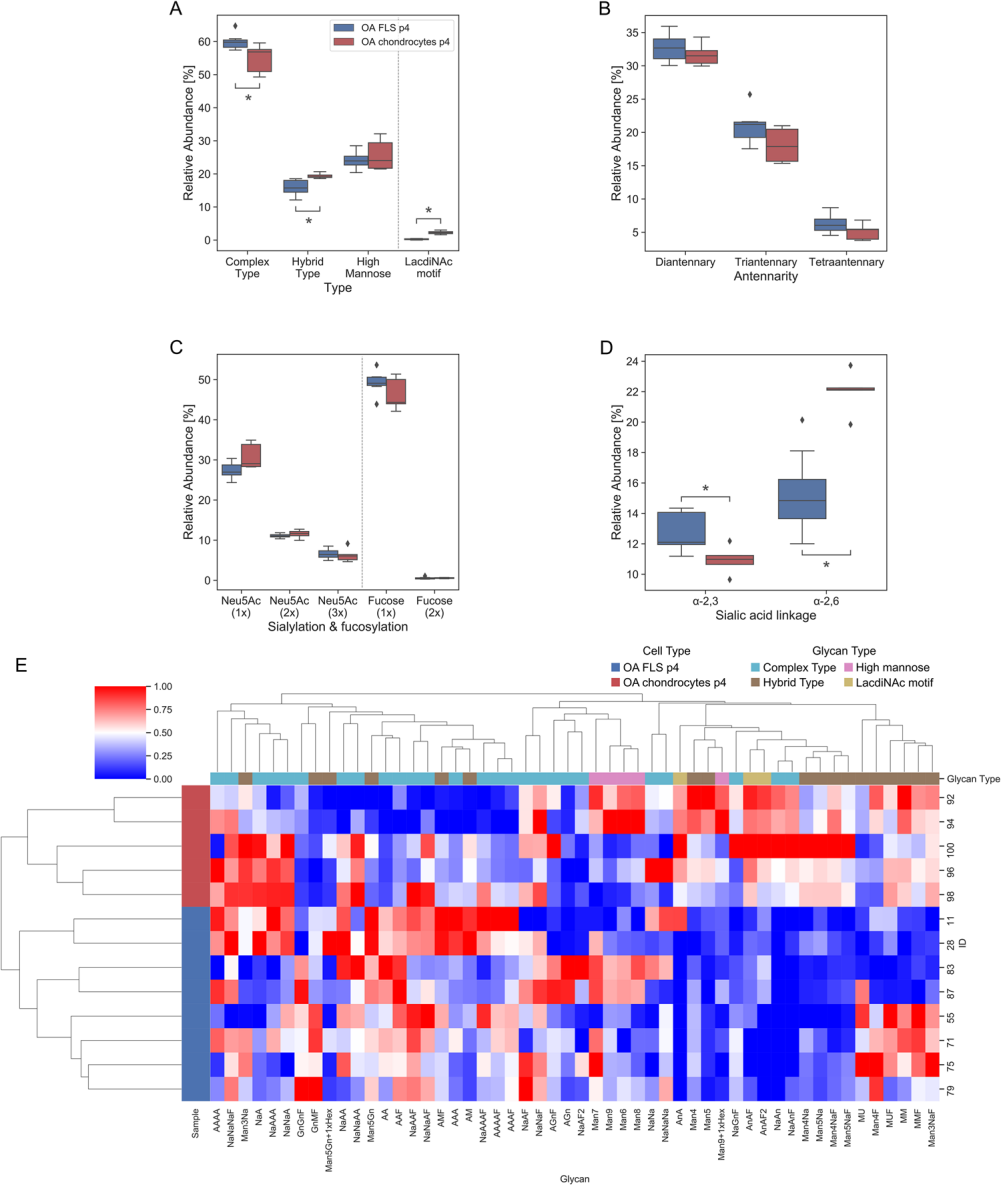
Comparison between the glycophenotypes of OA FLS and OA chondrocytes at p4. (A‐D) Box plots show the relative N‐glycan abundance found in OA FLS (n = 8 patients) and OA chondrocytes at p4 (n = 5 patients), grouped according to (A) types of N‐glycans (B) antennarity of complex‐type structures (C) frequency of sialylation and fucosylation, and (D) sialic acid linkage. The cell type is assigned to the boxes by color coding according to the inset in panel (B). Significant differences between groups are indicated with asterisks (*p < 0.05; unpaired *t*‐test or Mann‐Whitney test). (E) Heatmap showing the hierarchical clustering of the relative glycan abundance in OA FLS (n = 8 patients) and OA chondrocytes at p4 (n = 5 patients)

Resulting from experiments performed under identical analytical conditions, the data on the N‐glycan profile of OA FLS established the basis for comparisons to those of OA chondrocytes (at p0 and p4). Detailed information about the samples is found in Table S2, the corresponding raw data are listed in Tables S3 (area under the curve) and S4 (retention time). In total, 147 N‐glycan structures, listed in Table S5, were detected and assigned to the different categories, that is, type of N‐glycan, number of antennae in complex‐type N‐glycans, number of sialic acid and fucose moieties, respectively, as well as linkage type of sialylation. In comparison, the FLS data show fewer cases of deviation from p4 OA chondrocytes (Figure 2A–D) than from p0 OA chondrocytes (Figure S2A‐D). Differences in the presence of LacdiNAc and α2,6‐sialylated branch ends between OA FLS and p0 OA chondrocytes appear to be reconcilable with RT‐qPCR data on the respective glycosyltransferases (Table S6). The respective heatmaps presented in Figure 2E and S2E give an overview on the level of different isobaric N‐glycan structures. Taken together, these data suggest an inherent difference in the N‐glycan profile between OA FLS and OA chondrocytes. This difference is diminished by passaging of chondrocytes that leads to morphological resemblance with OA FLS.

To allow comparison of data from patient‐derived OA FLS with a standardized cell line, immortalized synovial fibroblasts K4IM were included into the analysis. Comparative analyses disclosed a number of differences such as an increased ratio between complex‐ and hybrid‐type structures or higher levels of triantennary structures in K4IM cells (Figure S3). Next, we aimed to examine the extent of susceptibility of N‐glycosylation in OA FLS to the presence of functional disease markers (IL‐1β, TNF‐α), in order to delineate the influence of a pro‐inflammatory microenvironment in situ.

### 3.3 Cytokines as modulators of OA FLS N‐glycosylation

**FIGURE 3:**
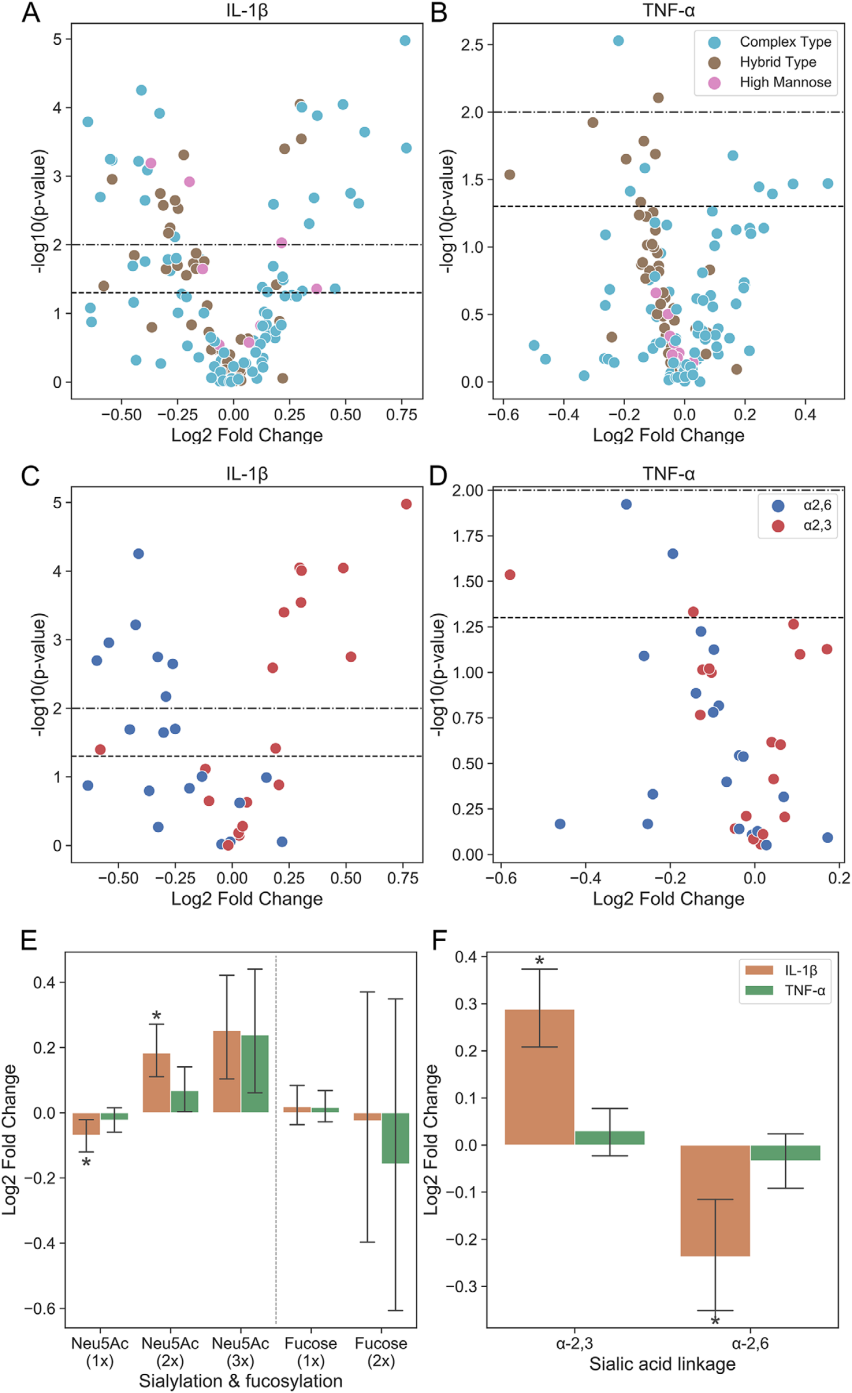
Impact of IL‐1β or TNF‐α on the abundance of N‐glycan structures in OA FLS. (A‐D) Volcano plot of the mean fold change (log2) of glycan representation between cytokine‐treated and untreated OA FLS versus the significance level (‐log10) of this difference after (A,C) IL‐1β or (B,D) TNF‐α treatment. The dots represent the results of paired samples from different patients (n = 14) and the type of N‐glycan (A,B) or of linkage in the sialylgalactose terminus (C,D) is indicated by color according to the inset in panel (B,D). *p*‐Values were calculated using the paired *t*‐test. The dashed horizontal lines indicate the *p*‐values of 0.05 and 0.01, respectively. (E‐F) Log2 fold change of distinct N‐glycan characteristics, including (E) extent of sialylation and fucosylation or (F) type of linkage of sialylgalactose, in OA FLS after treatment with IL‐1β or TNF‐α in comparison to paired controls (n = 14 patients). The type of cytokine treatment is indicated by color according to the inset in panel (D). Significant differences to the untreated controls are indicated with asterisks (*p < 0.05; n = 14; paired two‐sided *t*‐test or Wilcoxon test)

Initial evidence for a modulation of N‐glycosylation by a pro‐inflammatory cytokine (IL‐1β) was collected by RT‐qPCR measurements (Table S7). These results suggested effects on i) sialylation by ST6GAL1 and ST3GAL4, ii) branching via MGAT4/5B and, most markedly, iii) N‐glycan maturation by MAN1C1. Detailed MS‐based characterization of N‐glycans in paired samples of 14 patients revealed that IL‐1β led to N‐glycan remodeling (affecting a total of 27 cases of N‐glycans after Benjamini‐Hochberg correction), stronger so than TNF‐α (Figure 3A,B). Fittingly, the shift from α2,6‐ to α2,3‐sialyation, induced by IL‐1β but not by TNF‐α (Figure 3C–F), was in line with the detected changes of ST6GAL1 and ST3GAL4 mRNA levels. Decrease in α2,6‐sialylation is a favorable factor for galectin binding, as is increase in α2,3‐sialylation especially for Gal‐8, posing the question on actual responsiveness of OA FLS to galectin binding. In order to provide first evidence for an active role of FLS in the response profile to galectin presence in situ, the status of expression of a functional disease marker upon exposure to selected galectins was determined.

### 3.4 Detection of elicitor activity of‐ galectins on FLS

**FIGURE 4:**
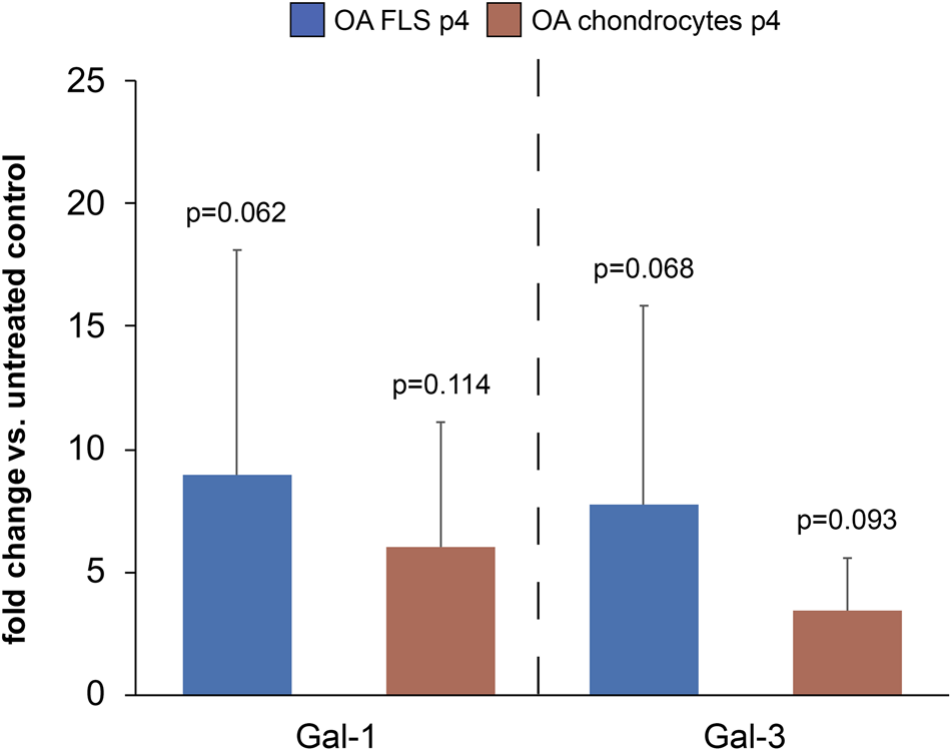
Effect of Gal‐1 and Gal‐3 on IL1B mRNA levels in OA FLS and OA chondrocytes at p4. Cell cultures of OA FLS, established from tissues of OA patients (n = 5), and of OA chondrocytes p4 (n = 3) were treated for 24 h with 10 μg/mL Gal‐1 or Gal‐3. Fold changes of IL1B mRNA levels (normalized to GAPDH) were evaluated using RT‐qPCR with respect to untreated control cells set to 1. *p*‐Values from the comparison to the untreated control given (paired, one‐sided *t*‐test)

The level of gene expression for IL1B was used as a sensor for respective galectin activity. Figure 4 shows that Gal‐1 and ‐3 upregulated this parameter in OA FLS, and that the measured effect was comparable to that in OA chondrocytes at p4. Primary cultures showed an enhanced level of activity that declined during the following steps of passaging (Figure S4).

## 4 DISCUSSION

N‐Glycans are a highly versatile means to dynamically fine‐tune the communication between cells and their environment [2, 23, 24]. Local density and the structures of the branch ends are factors that commonly specify their recognition by lectins. Concerning human galectins, which are multifunctional effectors triggering a host of clinically relevant outside‐in signaling [25, 26, 27, 28], the number of antennae in the complex‐type category, status and linkage type of sialylation and also LacdiNAc presence can modulate the interaction [29, 30]. For example, positioned at an early stage in N‐glycan maturation, the Golgi α1,2‐mannosidase I (MAN1C1) is the control point for conversion of high‐mannose‐ to hybrid‐type N‐glycans. An inhibition at this site can not only affect correct routing but also galectin‐dependent lattice formation of glycoproteins, as exemplified for basigin (CD147), an inducer of matrix metalloproteinases [31, 32], and Gal‐3 [33, 34]. In turn, this galectin is a receptor for LacdiNAc [35, 36]. Also presented at terminal positions, N‐acetyllactosamine (LacNAc) made accessible by reduction of extent of α2,6‐sialylation is a growth‐regulatory signal in activated T and carcinoma cells ‘read’ by Gal‐1 [37, 38], whereas α2,3‐sialylated structures bind Gal‐8, a potent pro‐ and anti‐inflammatory mediator [39], with nM affinity [40]. Such examples for an interplay illustrate the potential for a functional meaning of shifts in the glycophenotype, here assessed by MS‐based profiling.

Our study has first added N‐glycosylation to the list of changes during dedifferentiation in the course of chondrocyte passaging, that is, their conversion to a fibroblast‐like phenotype. So far, shifts in gene expression upon dedifferentiation of articular chondrocytes have predominantly been attributed to matrix proteins, most prominently to the switch from type II to type I collagen, proteinases and cytokines [12, 13, 41, 42, 43].

The determination of the N‐glycan profile in OA FLS enabled us to answer the question on a relationship to p4 chondrocytes and immortalized FLS. Obviously, there are similarities in the N‐glycome between morphologically similar cell types. The deserved deviations between the cell line and clinical (OA) material support the preference for material obtained from surgical specimen for experimental studies. The detected responsiveness of OA FLS to galectins (especially in primary cultures) and their susceptibility to attain altered N‐glycosylation in a pro‐inflammatory microenvironment put OA FLS into focus for upcoming systematic elicitor testing with galectins. Of note, these results encourage to proceed to work with OA FLS at p0, inevitably available in small quantities. In analogy to chondrocytes, the identification of OA FLS glycoproteins acting as galectin counterreceptors and of the actual contact sites on the level of glycans will not only provide clues on signaling routes during pathogenesis but also define distinct targets for blocking this clinically unfavorable pairing. Of note, the example of enhanced chemokine production by a galectin (i.e., Gal‐8), for example CXCL12 (a binding partner of Gal‐3 by interaction with its F‐face [44]) involving also protein recognition in osteoblasts [45], advises to consider such possibilities, too. Successful blocking of galectin binding to cell surfaces by bioactive peptides from the carbohydrate recognition domains of galectins [46] suggests the feasibility of devising such a type of competitive inhibitor, using the galectin as source.

In summary, this work underscores the non‐uniform nature of events that regulate N‐glycan presentation and defines this feature for OA FLS. The data further suggest to examine the potential of lectins to selectively manipulate glycoprotein function in chronic inflammatory disease with therapeutic intention, as recently outlined [47]. A certainly ambitious aim is to find ways to interfere with disease progression by, for example, blocking galectin‐glycoprotein pairing and manipulating in situ glycosylation [48], as recently also suggested in the case of coronaviral infection by their galectin‐like adhesins [49]. In this context, insights into glycome representation may offer inspiration for innovations to master the enormous challenge of finding new treatment modalities for OA [50].

## CONFLICT OF INTEREST

The authors have declared no conflict of interest.

## Supporting information

SUPPORTING INFORMATION
