# Supplementary material for: N‐Glycan profiling of chondrocytes and fibroblast‐like synoviocytes: Towards functional glycomics in osteoarthritis
Source: Proteomics Clin Appl. 2021 Mar 12;15(2-3):2000057. doi: 10.1002/prca.202000057 (PMC8548877; doi:10.1002/prca.202000057)
Supplement: Supplementary file 1 — SUPPORTING INFORMATION [file PRCA-15-2000057-s001.docx]

## Supplementary Table S1

## Presence of mRNA for selected N-glycan glycosyltransferases in OA chondrocytes in p0 and p4. Cell cultures of OA chondrocytes p0 were established from cartilage tissues from four patients, whereas OA chondrocytes p4 were generated by subsequent passaging of the same cultures. Distinct mRNA species were quantified using RT-qPCR. Numbers denote relative copy numbers (mean ± standard deviation) with respect to the geometric mean of the expression of GAPDH, ACTB and SDHA arbitrarily set to 1000. p-Values resulting from the analysis with the paired two-sided t-test are given. Bold p-values indicate results of p<0.05.

|  | **MAN1C1** | **MAN2A1** | **MGAT1** | **MGAT2** | **MGAT3** | **MGAT4A** | **MGAT4B** | **MGAT5A** |
| --- | --- | --- | --- | --- | --- | --- | --- | --- |
| **OA chondrocytes p0** | 1.1±1.1 | 155.3±31.5 | 1750.4±387.7 | 1011.0±271.7 | 13.5±13.7 | 79.4±45.6 | 117.2±23.0 | 104.0±40.9 |
| **OA chondrocytes p4** | 1.7±0.8 | 116.2±30.6 | 1461.6±286.6 | 804.2±225.4 | 11.3±6.8 | 22.5±19.5 | 234.2±37.7 | 107.7±42.0 |
| **p-value** | 0.565 | 0.192 | 0.256 | 0.310 | 0.831 | **0.042** | **0.007** | 0.919 |

|  | **MGAT5B** | **FUT8** | **B4GALNT3** | **ST6GAL1** | **ST6GAL2** | **ST3GAL3** | **ST3GAL4** | **ST3GAL6** |
| --- | --- | --- | --- | --- | --- | --- | --- | --- |
| **OA chondrocytes p0** | 2.4±0.7 | 148.6±29.5 | 114.1±22.1 | 934.8±374.2 | 2.3±0.6 | 74.5±13.7 | 211.6±65.2 | 25.3±5.7 |
| **OA chondrocytes p4** | 5.9±4.7 | 206.8±22.3 | 64.3±20.9 | 624.1±363.3 | 34.5±36.4 | 61.9±7.6 | 290.3±147.0 | 48.3±17.9 |
| **p-value** | 0.196 | 0.064 | **0.003** | 0.197 | 0.171 | 0.248 | 0.322 | 0.055 |

## Supplementary Figure S1

Cell cultures of OA chondrocytes p0 and OA chondrocytes p4, established from cartilage tissues of three patients, were treated for 24 h with 10 µg/ml and 50 µg/ml Gal-1 or Gal-3. Fold changes (log10) of IL1B mRNA levels (normalized to GAPDH) were evaluated using RT-qPCR with respect to untreated control cells set to 1. Significant differences between groups are indicated with asterisks (*p<0.05; n=3; paired one-sided t-test).

## Supplementary Table S2

List of all samples analyzed in this work including the type of cells, the patient number, the number of passages for the cell culture and the treatment. Each individual sample was given a unique number for identification (ID). The treatment option ‘Control’ indicates untreated cells.

| **ID** | **Cell Type** | **Patient No.** | **Passage Number** | **Treatment** |
| --- | --- | --- | --- | --- |
| **9** | OA Chondrocytes | 1204 | 0 | Control |
| **15** | OA Chondrocytes | 1217 | 0 | Control |
| **32** | OA Chondrocytes | 1233 | 0 | Control |
| **37** | OA Chondrocytes | 1247 | 0 | Control |
| **41** | OA Chondrocytes | 1248 | 0 | Control |
| **43** | OA Chondrocytes | 1250 | 0 | Control |
| **59** | OA Chondrocytes | 1260 | 0 | Control |
| **67** | OA Chondrocytes | 1265 | 0 | Control |
| **91** | OA Chondrocytes | 1359 | 0 | Control |
| **92** | OA Chondrocytes | 1359 | 4 | Control |
| **93** | OA Chondrocytes | 1364 | 0 | Control |
| **94** | OA Chondrocytes | 1364 | 4 | Control |
| **95** | OA Chondrocytes | 1373 | 0 | Control |
| **96** | OA Chondrocytes | 1373 | 4 | Control |
| **97** | OA Chondrocytes | 1380 | 0 | Control |
| **98** | OA Chondrocytes | 1380 | 4 | Control |
| **99** | OA Chondrocytes | 1392 | 0 | Control |
| **100** | OA Chondrocytes | 1392 | 4 | Control |
| **36** | K4IM | NA | 36 | Control |
| **45** | K4IM | NA | 37 | Control |
| **46** | K4IM | NA | 38 | Control |
| **1** | OA FLS | 1172 | 4 | Control |
| **2** | OA FLS | 1172 | 4 | IL-1β |
| **3** | OA FLS | 1172 | 4 | TNF-α |
| **5** | OA FLS | 1176 | 4 | Control |
| **6** | OA FLS | 1176 | 4 | IL-1β |
| **7** | OA FLS | 1176 | 4 | TNF-α |
| **11** | OA FLS | 1204 | 4 | Control |
| **12** | OA FLS | 1204 | 4 | IL-1β |
| **13** | OA FLS | 1204 | 4 | TNF-α |
| **16** | OA FLS | 1214 | 4 | Control |
| **17** | OA FLS | 1214 | 4 | IL-1β |
| **18** | OA FLS | 1214 | 4 | TNF-α |
| **20** | OA FLS | 1215 | 4 | Control |
| **21** | OA FLS | 1215 | 4 | IL-1β |
| **22** | OA FLS | 1215 | 4 | TNF-α |
| **24** | OA FLS | 1216 | 4 | Control |
| **25** | OA FLS | 1216 | 4 | IL-1β |
| **26** | OA FLS | 1216 | 4 | TNF-α |
| **28** | OA FLS | 1217 | 4 | Control |
| **29** | OA FLS | 1217 | 4 | IL-1β |
| **30** | OA FLS | 1217 | 4 | TNF-α |
| **51** | OA FLS | 1232 | 4 | Control |
| **52** | OA FLS | 1232 | 4 | IL-1β |
| **53** | OA FLS | 1232 | 4 | TNF-α |
| **55** | OA FLS | 1233 | 4 | Control |
| **56** | OA FLS | 1233 | 4 | IL-1β |
| **57** | OA FLS | 1233 | 4 | TNF-α |
| **71** | OA FLS | 1247 | 4 | Control |
| **72** | OA FLS | 1247 | 4 | IL-1β |
| **73** | OA FLS | 1247 | 4 | TNF-α |
| **75** | OA FLS | 1248 | 4 | Control |
| **76** | OA FLS | 1248 | 4 | IL-1β |
| **77** | OA FLS | 1248 | 4 | TNF-α |
| **79** | OA FLS | 1250 | 4 | Control |
| **80** | OA FLS | 1250 | 4 | IL-1β |
| **81** | OA FLS | 1250 | 4 | TNF-α |
| **83** | OA FLS | 1260 | 4 | Control |
| **84** | OA FLS | 1260 | 4 | IL-1β |
| **85** | OA FLS | 1260 | 4 | TNF-α |
| **87** | OA FLS | 1265 | 4 | Control |
| **88** | OA FLS | 1265 | 4 | IL-1β |
| **89** | OA FLS | 1265 | 4 | TNF-α |

## Supplementary Table S3

Table listing the AUC (Area Under the Curve) for each identified glycan in every sample that was analyzed. Glycan ID is based on Supplementary Table S5 and the sample ID is referenced in Supplementary Table S4. A value of 0 indicates that the glycan was not detected in this sample.

*(please see separate file)*

## Supplementary Table S4

Table listing retention time in minutes for each identified glycan in every sample that was analyzed. Glycan ID is based on Supplementary Table S5 and the sample ID is referenced in Supplementary Table S4. An empty cell indicates that the respective glycan was not detected in this sample and thus no retention time was determined.

*(please see separate file)*

## Supplementary Table S5

List of all glycans analyzed during this study, including their mass and sugar composition. The structure of each glycan was inferred from its mass and fragmentation pattern. Glycoforms of each glycan were distinguished based on the elution profile. Each glycan was given a unique ID. The average retention time in minutes is given. The detailed values for each glycan in each measurement can be found in Supplementary Table S7.

*(please see separate file)*

## Supplementary Figure S2

Comparison between the glycophenotypes of OA FLS and OA chondrocytes. Cell cultures of OA FLS and OA chondrocytes p0 were established from synovial and cartilage tissues, respectively, collected from the same patients (n=8). **(A-D)** Box plots show the relative glycan abundance in OA FLS and OA chondrocytes p0 (n=8 patients), grouped according to **(B)** types of glycans, **(C)** antennarity of complex type structures, **(D)** prevalence of sialylation and fucosylation, and **(E)** sialic acid linkage. The cell type is assigned to the boxes by color coding according to the inset in panel (B). Significant differences between groups are indicated with asterisks (*p<0.05; n=8; paired two-sided t-test or Wilcoxon test). **(E)** Heatmap showing the hierarchical clustering of standardized glycan abundance based on a Euclidean distance matrix.


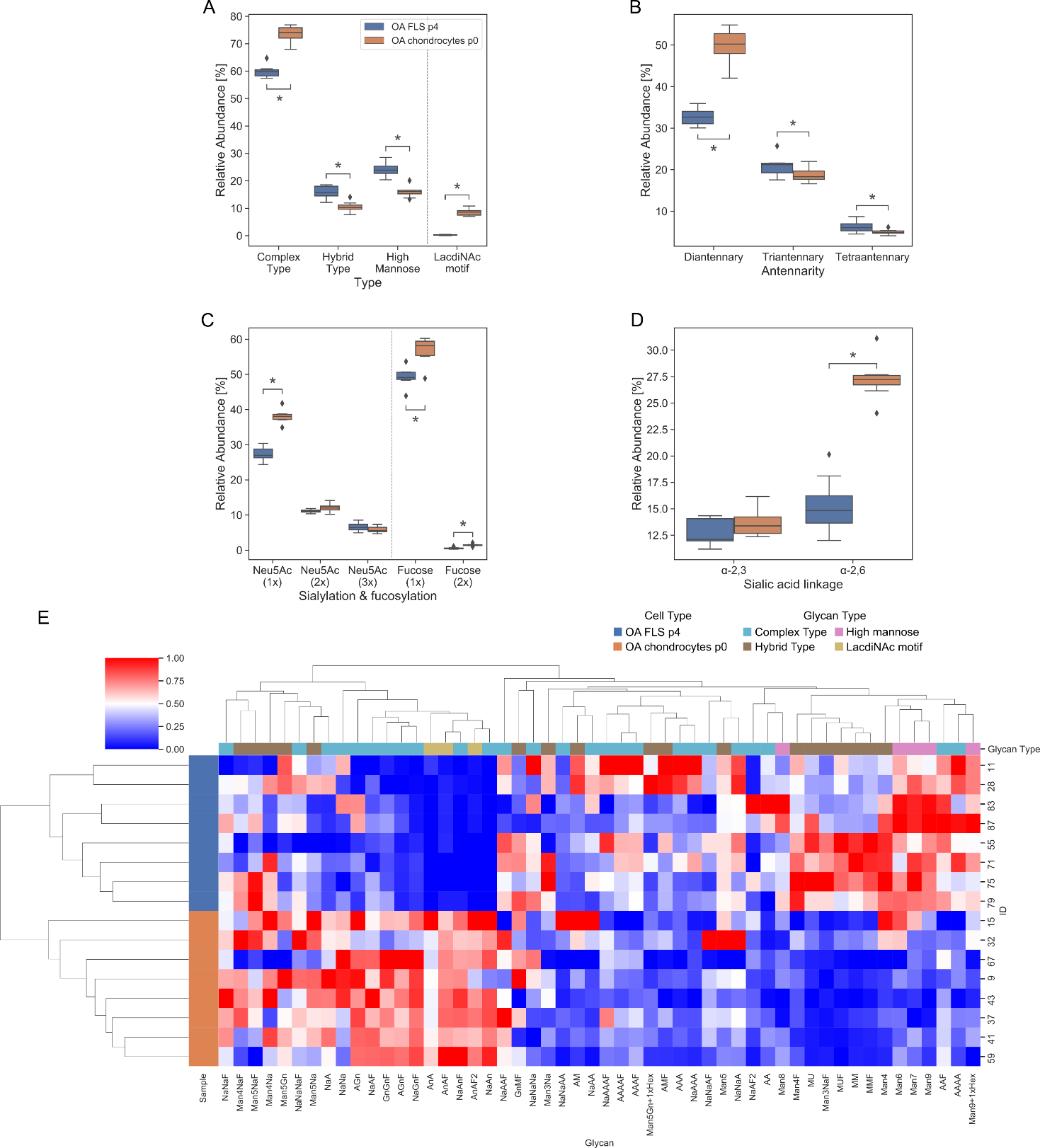


## Supplementary Table S6

## Presence of mRNA for selected N-glycan glycosyltransferases in OA FLS and OA chondrocytes. Cell cultures of OA FLS and OA chondrocytes p0 were established from synovial and cartilage tissues, respectively, collected from the same patients (n=4). Distinct mRNA species were quantified using RT-qPCR. Numbers denote relative copy numbers (mean ± standard deviation) with respect to the geometric mean of the expression of GAPDH, ACTB and SDHA arbitrarily set to 1000. p-Values resulting from the analysis with the paired two-sided t-test are given. Bold p-values indicate results of p<0.05.

|  | **MAN1C1** | **MAN2A1** | **MGAT1** | **MGAT2** | **MGAT3** | **MGAT4A** | **MGAT4B** | **MGAT5A** |
| --- | --- | --- | --- | --- | --- | --- | --- | --- |
| **OA FLS** | 1.5±0.5 | 339.2±0.7 | 2381.1±793.6 | 752.9±197.8 | 1.4±0.8 | 13.1±8.5 | 242.4±53.0 | 86.7±40.6 |
| **OA chondrocytes** | 0.5±0.5 | 140.0±43.6 | 1615.7±273.1 | 2303.3±3130.1 | 4.4±2.2 | 53.6±16.0 | 125.4±17.7 | 85.2±17.8 |
| **p-value** | **0.002** | 0.134 | 0.099 | 0.376 | 0.112 | **0.003** | **0.028** | 0.960 |

|  | **MGAT5B** | **FUT8** | **B4GALNT3** | | **ST6GAL1** | **ST6GAL2** | **ST3GAL3** | **ST3GAL4** | **ST3GAL6** |
| --- | --- | --- | --- | --- | --- | --- | --- | --- | --- |
| **OA FLS** | 5.4±3.3 | 603.2±193.0 | 3.0±5.9 | | 158.4±115.3 | 2.5±1.9 | 89.0±45.8 | 202.1±47.2 | 40.8±25.3 |
| **OA chondrocytes** | 2.6±1.5 | 236.5±22.3 | 120.4±23.9 | | 933.6±184.5 | 5.7±2.4 | 78.4±18.5 | 167.3±27.1 | 47.9±19.3 |
| **p-value** | 0.131 | **0.025** | | **0.002** | **0.004** | 0.206 | 0.717 | 0.158 | 0.614 |

## Supplementary Figure S3

Characterization of the OA FLS glycophenotype in comparison to the glycophenotype of K4IM cells. Shown is the relative glycan abundance found in OA FLS (n=14 patients) and K4IM cells (n=3), grouped according to **(A)** types of glycans, **(B)** antennarity of complex type structures, and **(C)** prevalence of sialylation and fucosylation. Dots represent the results from different patients or K4IM cells from different passages. Lines indicate the median for each category. Significant differences between groups are indicated with asterisks (*p<0.05; unpaired two-sided t-test or Mann-Whitney test). **(E)** Heatmap showing the hierarchical clustering of standardized glycan abundance based on a Euclidean distance matrix.


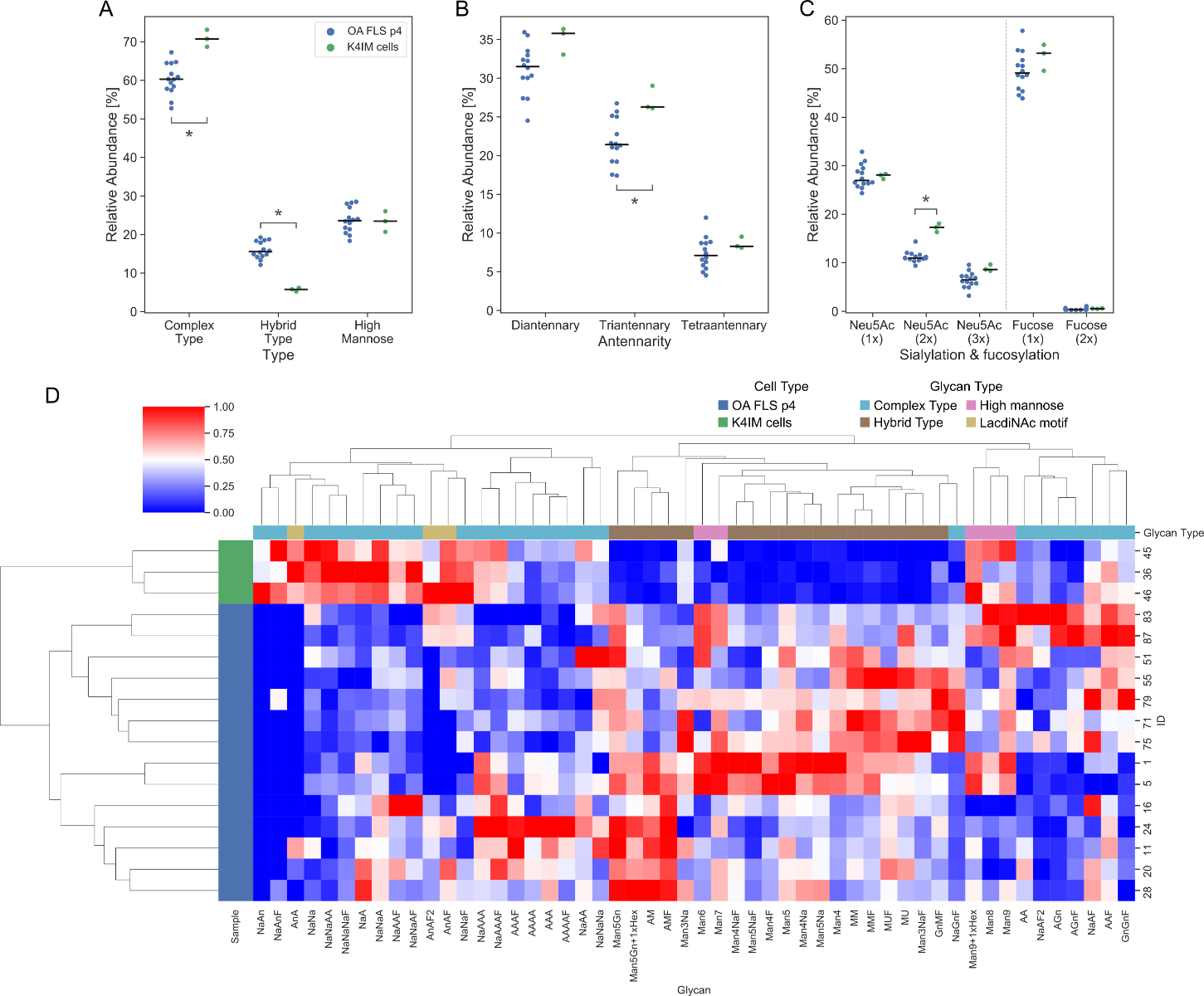


## Supplementary Table S7

## Effect of IL-1β on the mRNA levels of selected N-glycan glycosyltransferases in OA FLS. Distinct mRNA species were quantified using RT-qPCR considering normalization to GAPDH as reference gene. Numbers denote mean fold changes (± standard deviation) induced by IL-1β in OA FLS from five donors. p-Values resulting from the analysis with the paired two-sided t-test are given. Bold p-values indicate results of p<0.05.

|  | **MAN1C1** | **MAN2A1** | **MGAT1** | **MGAT2** | **MGAT3** | **MGAT4A** | **MGAT4B** | **MGAT5A** |
| --- | --- | --- | --- | --- | --- | --- | --- | --- |
| **Fold change** | 0.4±0.1 | 1.3±0.7 | 1.5±0.7 | 3.1±1.4 | 0.7±0.2 | 0.5±0.1 | 0.7±0.3 | 1.7±0.7 |
| **p-value** | **0.0003** | 0.493 | 0.259 | 0.089 | 0.080 | **0.008** | **0.028** | 0.278 |

|  | **MGAT5B** | **FUT8** | **B4GALNT3** | **ST6GAL1** | **ST6GAL2** | **ST3GAL3** | **ST3GAL4** | **ST3GAL6** |
| --- | --- | --- | --- | --- | --- | --- | --- | --- |
| **Fold change** | 11.7±7.1 | 0.8±0.5 | 6.3±4.0 | 0.8±0.1 | 2.8±0.9 | 1.4±0.4 | 4.7±1.7 | 0.7±0.3 |
| **p-value** | **0.041** | 0.237 | 0.109 | **0.042** | 0.081 | 0.226 | **0.048** | 0.065 |

## Supplementary Figure S4

Effect of Gal-1 and Gal-3 on IL1B mRNA levels in OA FLS over repeated passages. Cell cultures of OA FLS were established from tissues of OA patients (n=3) and passaged until p4. In each passage, cells were treated for 24 h with 10 µg/ml Gal-1 or Gal-3, or were left untreated. Fold changes (log) of IL1B mRNA levels (normalized to GAPDH) were evaluated using RT-qPCR with respect to untreated control cells set to 1. Significant differences to the untreated controls of the respective passage are indicated with asterisks (*p<0.05; paired, one-sided t-test). P-Values between 0.05 and 0.1 are given in numbers.
